# A Two-Stage Lightweight Deep Learning Framework for Mass Detection and Segmentation in Mammograms Using YOLOv5 and Depthwise SegNet

**DOI:** 10.1007/s10278-025-01471-0

**Published:** 2025-03-14

**Authors:** Dimitris Manolakis, Paschalis Bizopoulos, Antonios Lalas, Konstantinos Votis

**Affiliations:** Information Technologies Institute, Centre for Research & Technology- Hellas, 6th km Harilaou - Thermis, Thermi, 57001 Greece

**Keywords:** Deep learning, Medical imaging, Breast cancer, Segmentation

## Abstract

Ensuring strict medical data privacy standards while delivering efficient and accurate breast cancer segmentation is a critical challenge. This paper addresses this challenge by proposing a lightweight solution capable of running directly in the user’s browser, ensuring that medical data never leave the user’s computer. Our proposed solution consists of a two-stage model: the pre-trained nano YoloV5 variation handles the task of mass detection, while a lightweight neural network model of just 20k parameters and an inference time of 21 ms per image addresses the segmentation problem. This highly efficient model in terms of inference speed and memory consumption was created by combining well-known techniques, such as the SegNet architecture and depthwise separable convolutions. The detection model manages an mAP@50 equal to 50.3% on the CBIS-DDSM dataset and 68.2% on the INbreast dataset. Despite its size, our segmentation model produces high-performance levels on the CBIS-DDSM (81.0% IoU, 89.4% Dice) and INbreast (77.3% IoU, 87.0% Dice) dataset.

## Introduction

Breast cancer is one of the most common types of cancer in women; 30% of all new female cancers are of this type in the USA, with the American Cancer Society estimating a total of 43,700 deaths from breast cancer in the USA in 2023 [1]. However, effective screening tests such as mammography can lead to a detection of breast tumors in an early stage, with many studies highlighting the importance of regular screening mammography especially for women older than 40 [2]. Breast cancer screening has its own risks, for instance, diagnosing tumors that would not be life-threatening (over-diagnosis) and possible false-positive test results, which lead to anxiety and follow-up test procedures [3]. Therefore, developing tools that aid doctors and radiology experts in detecting and segmenting tumors can be very beneficial in reducing diagnostic errors in mammography. Consequently, a significant number of computer-aided detection (CAD) systems have been developed with the aim of enhancing detection accuracy [4–7].

Mass segmentation is a key feature of CAD systems, as the presence of breast mass is a sign of cancer. Besides detecting a mass, extracting the shape and margin of breast masses is necessary in order to determine their malignancy; irregular shapes suggest a higher likelihood of malignancy [8]. Yet mass segmentation is a difficult task due to indistinctive boundaries of masses. Furthermore, manual screening of mammograms can be quite costly and time-consuming [9], while traditional segmentation methods require hand-crafted features and the optimization of many parameters and thresholds, leading to unstable results [6].

The resurgence of neural networks gave way to the application of deep learning (DL) techniques in the field of medical imaging. Deep convolutional neural networks (CNN) are one of the dominant techniques in the field of computer vision, due to their ability to efficiently process and model images, learning spatial hierarchies of features through multi-layer network structures [10]. They have been widely used with the aim of extracting meaningful features from mammograms, as well as classifying detected tumors as benign or malignant [11, 12]. Later on, U-Net was developed specifically for the task of biomedical image segmentation [13]. It is based on CNN, using a encoder-decoder architecture in order to produce a segmentation map based on the input image, localizing and distinguishing borders by classifying each pixel. A distinctive feature of this architecture is the use of skip connections that concatenate feature maps of the encoder with the corresponding ones in the decoder. SegNet [14] is another encoder-decoder architecture based on CNN that is efficient in terms of memory and computational time, due to the fact that it only stores the max-pooling indices of the features maps, utilizing them in the decoder network.

In this paper, we present a lightweight neural network inspired by the SegNet architecture, coupled with other well-known efficient techniques such as depthwise convolutional layers. The primary objective is to achieve good performance while minimizing computational costs. This efficiency facilitates the integration of our model into a web application running in the user’s browser, leveraging frameworks like TensorFlow.js [15].

**Fig. 1:**
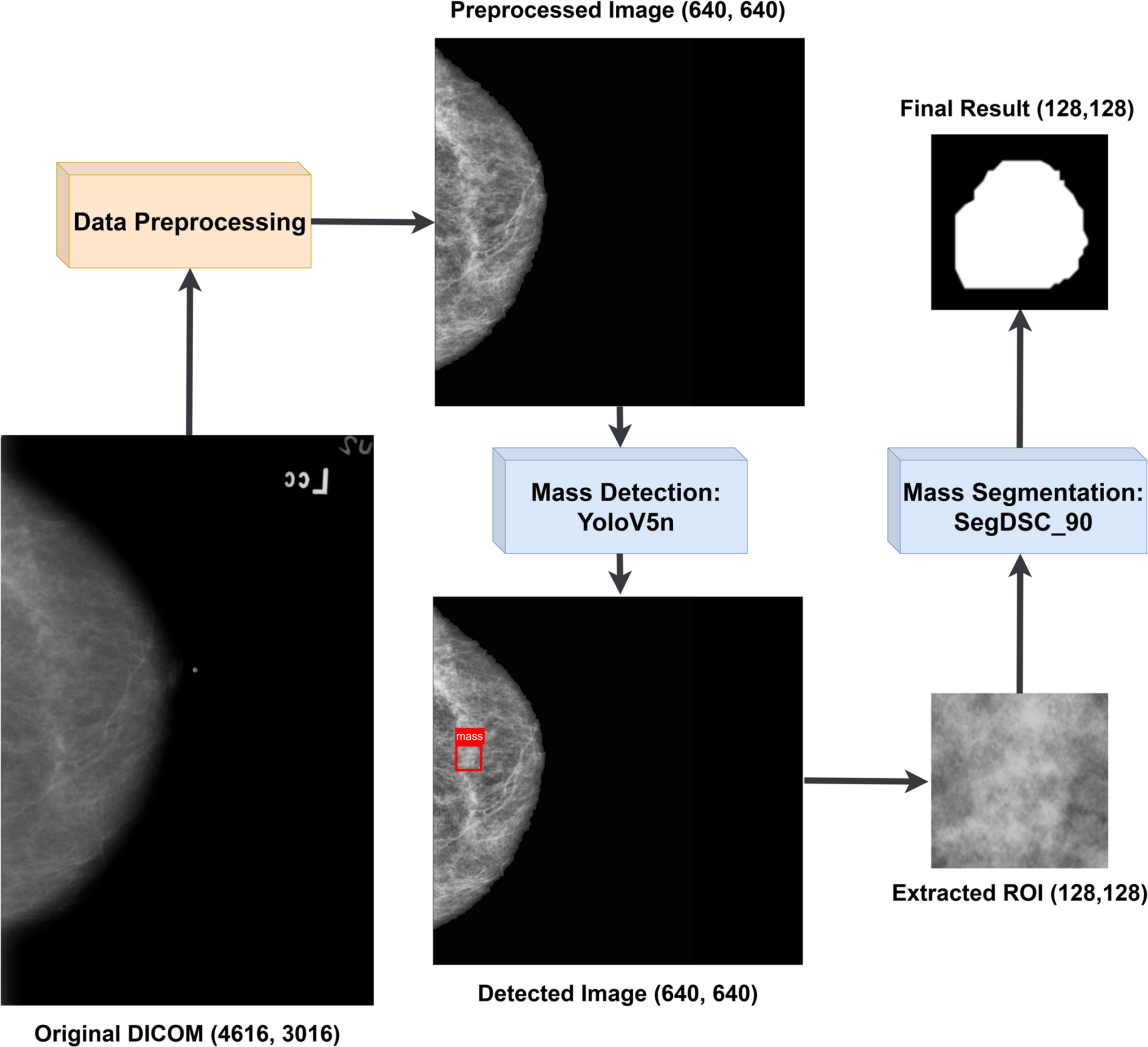
Architecture of the proposed lightweight two-stage model: Mammograms are first preprocessed and then fed into the YOLOv5n model for mass detection, generating bounding boxes. Regions of interest are then extracted using the bounding boxes and processed by the SegDSC_90 model for mass segmentation

Considering the fact that breast masses usually only appear in localized areas, it is necessary to implement detection models that are able to pinpoint regions of interest (ROIs) which can then be given as input to the segmentation models. Consequently, we developed a two-stage model that solves both the mass detection and segmentation problems. For the task of mass detection on mammograms, we opted for transfer learning using state-of-the-art detection models YoloV5 and YoloV8, focusing on optimizing the performance of the smaller and faster versions such as YoloV5n6 and YoloV8n. Our approach aims to enhance the overall effectiveness of our model in real-world applications. Figure 1 depicts the architecture of the proposed two-stage model, outlining the preprocessing, detection, and segmentation pipeline.

The rest of the paper is organized as follows: Section “Related Work” presents a review of the background literature, whereas Section “Dataset” summarizes the used data preprocessing techniques. Section “Methodology” provides an overview of the applied methodologies, and Section “Experimental Results” presents the experimental results. Finally, Section “6” concludes the paper and poses future challenges.

## Related Work

In recent years, a significant amount of effort has been invested in the task of mass segmentation on mammograms, with many works presenting surveys on the subject [16, 17]. The various methods used for this task can be classified as machine learning and deep learning segmentation, as well as classic segmentation (edge-based, threshold-based, and region-based) [17]. Petrick et al. [18] developed a region-growing technique, utilizing a density-weighted contrast enhancement filter as a pre-processing step. Their implementation managed to detect 98% of the biopsy-proven breast masses in a dataset of 253 mammograms. In [19], a region-growing algorithm is used in order to detect ROIs on mammograms, while the mass is then segmented using OTSU segmentation (threshold-based) [20]. The implementation was tested on a dataset of 485 mammograms containing 260 masses acquired from the Dokuz Eylul Mammography Set and achieved an accuracy of 95.06%.

As regards machine learning methods, Hizukuri et al. [21] combined threshold-based and machine learning by using a grey-level thresholding technique for feature extraction of mammogram calcifications and an artificial neural network for classification. Evaluating on a dataset of 96 mammogram images, the implementation achieved a detection rate of 96.5% with a shape-segmentation accuracy of 91.4%. The authors of [22] developed an efficient segmentation method that utilizes structured support vector machine, achieving a Dice coeffiecient of 87%, in the public datasets Digital Database for Screening Mammography (DDSM) [23] and INbreast [24]. In [25], a hybrid of K-means and support vector machine algorithms was used in the task of mass segmentation. The K-means algorithm clusters tumors based on similar features associated with malignancy or benignity, and the extracted features are then classified using an SVM classifier. Finally, the K-SVM algorithm was tested on the WDBC [26] data using 10-fold cross validation achieving an accuracy of 97.38%.

U-Net models are highly effective for biomedical image segmentation, particularly when working with limited data [27]. Cho et al. [6] developed a U-Net architecture that was trained on 63 ROIs of INbreast mammograms. Using data augmentation to balance the small number of samples, they achieved an average Dice coefficient of 80%. In [28], the authors devised a U-Net model that used the VGG-16 [29] architecture for the encoder network, achieving an Intersection over Union (IoU) of 65.24% on the CBIS-DDSM dataset [30]. Baccouche et al. [7] proposed Connected-UNet, an architecture that connects two UNets using additional modified skip connections, while also integrating atrous spatial pyramid pooling [31]. The implementation was applied on Residual UNet [32] and evaluated on the INbreast dataset, achieving a Dice score of 94.13%. Li et al. [33] developed MA-UNet, integrating multi-scale features and a hybrid attention mechanism. The network employs a two-stage encoder for enhanced feature extraction, optimizes skip connections to reduce noise, and introduces a simple self-attention mechanism in the transformer bottleneck to improve long-range dependencies while lowering computational costs. Evaluated on three datasets spanning different imaging modalities, including CT, ultrasound, and retinal images, MA-UNet consistently outperformed or matched 10 state-of-the-art models across all benchmarks. Wang et al. [34] proposed CSAU-Net, a cross-scale attention-guided U-Net that combines convolutional neural networks and transformers to enhance breast ultrasound image segmentation. This model integrates a cross-scale cross-attention transformer block within U-Net’s skip connections to improve feature extraction and segmentation accuracy. Experimental results on three public datasets demonstrate that CSAU-Net outperforms state-of-the-art segmentation models, achieving a Dice score of 81.22%, a Jaccard index of 70.92%, and an accuracy of 95.53% on the BUSI dataset, surpassing previous methods such as TransUNet and DeepLabV3+.

Regarding the Yolo architecture, it has been used previously on the task of mass detection on mammograms, with the authors of [35] applying transfer learning on YoloV3 [36] and YoloV5 [37] pre-trained variants. It was shown that YoloV5 models outperform other detection techniques such as Faster R-CNN and Single-Shot detection, achieving an mAP of 65% on the CBIS-DDSM dataset. Du et al. [38] developed YOLO-CPC, an enhanced breast tumor detection model based on YOLOv7. The backbone integrates a convolutional block attention module to focus on tumor regions while suppressing background noise. The efficient layer aggregation network module incorporates partial convolutions to minimize redundant computations, and coordinate convolution is used in the network’s neck to improve positional awareness and multi-scale feature extraction. These enhancements enabled YOLO-CPC to achieve 97.01% accuracy, 97.98% recall, and 90.78% AP50, outperforming YOLOv7 and other models like Faster R-CNN, YOLOv3, YOLOv5, and YOLOv6.

Reducing the computational cost of medical deep learning models is an active area of research. For example, in [39], it was shown that lightweight convolutional neural networks can beat transfer learning in COVID-19 detection from chest X-ray images. Asif et al. [40] utilized feature fusion with MobileNet and MobileNetV2 to develop a lightweight medical classification model applicable to various medical imaging modalities. Regarding medical segmentation, MedSAM [41] represents a state-of-the-art model capable of segmenting images across 10 modalities and over 30 cancer types. The *Segment Anything in Medical Images on Laptop* challenge [42] aimed to develop a lightweight model with comparable performance that could run efficiently on a laptop. The winning team utilized the EfficientVit model to distill knowledge from the MedSAM model and re-implemented the inference pipeline in C++ to optimize runtime for edge devices.

## Dataset

In this paper, the curated breast imaging subset of DDSM (CBIS-DDSM) [30] was used, which contains a total of 2620 scanned film mammography images. Out of these mammograms, we opted to use only the ones containing mass abnormalities, excluding calcification abnormalities, creating a dataset of 1592 mammograms and 1592 binary masks, which provide the position of the abnormalities. The dataset was already split into training (1231 samples) and testing subsets (361 samples). Additionally, in order to evaluate the generalization of our models, we also used the INbreast dataset (107 mammograms plus their corresponding masks) as a separate testing set.

### Data Preprocessing

**Fig. 2:**
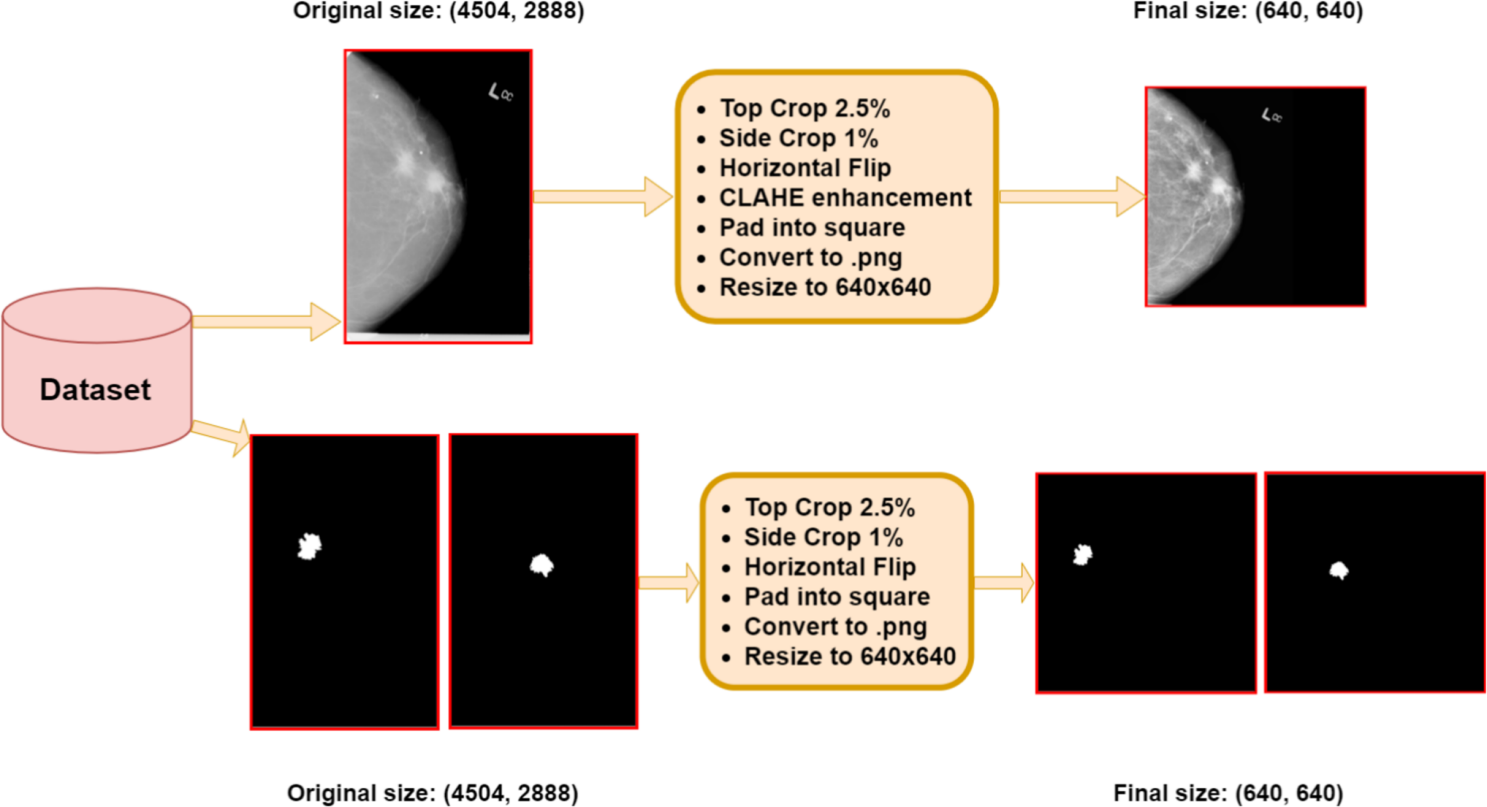
Initial step of the data preprocessing. Borders were cropped (2.5% top/bottom, 1% sides) to remove bright edges while preserving breast regions. All images were oriented to face right and processed with CLAHE to enhance grayscale details, improving model performance. Images were padded to squares, resized to 640x640 for Yolo training, and saved in PNG format with pixel values normalized to [0,255]

This section provides an overview of the data preprocessing steps used in this work. Figure 2 illustrates the primary phase of the preprocessing procedure that was applied to both the mammograms and the masks. Initially, most of the mammograms displayed bright white borders along their edges, which were removed by cropping the images. In accordance with previous studies [35], the ratio of the top and bottom image cropping was set to 2.5%, while the side crop ratio was set to just 1% in order to prevent unintentional removal of breast regions. The original datasets included mammograms imaged with different views, such as the mediolateral oblique view and cranial caudal view. Considering the fact that the majority of mammograms in the datasets were facing right, all mammograms (and their corresponding masks) exhibiting the opposite orientation are prepossessed to face right.

In the case of mammograms, we applied contrast limited adaptive histogram equalization (CLAHE) [43]. It is a preprocessing technique that has been shown to improve detection accuracy by observers [44] and has been used in previous mass segmentation works [7]. This method enhances the small details and features of greyscale images, which can significantly improve the models’ performance. Additionally, we considered the potential benefits of artefact removal [45] in order to remove non-informative noise (e.g., tags identifying the scan’s orientation), but after experimenting, we found that this technique did not improve performance. Hence, we opted to skip this preprocessing step.

Regarding size and aspect ratio, most images had different sizes and were not square. Consequently, all images are padded by adding black pixels into squares and had their size reduced to 640x640, which is the resolution employed for training the Yolo models featured in this paper. Finally, the pixel values of all images were converted to a range of [0,255] and stored in PNG format. Note that each mammogram in both the CBIS-DDSM and INbreast dataset may exhibit one or more masses, as illustrated in Fig. 2, for instance.

**Fig. 3:**
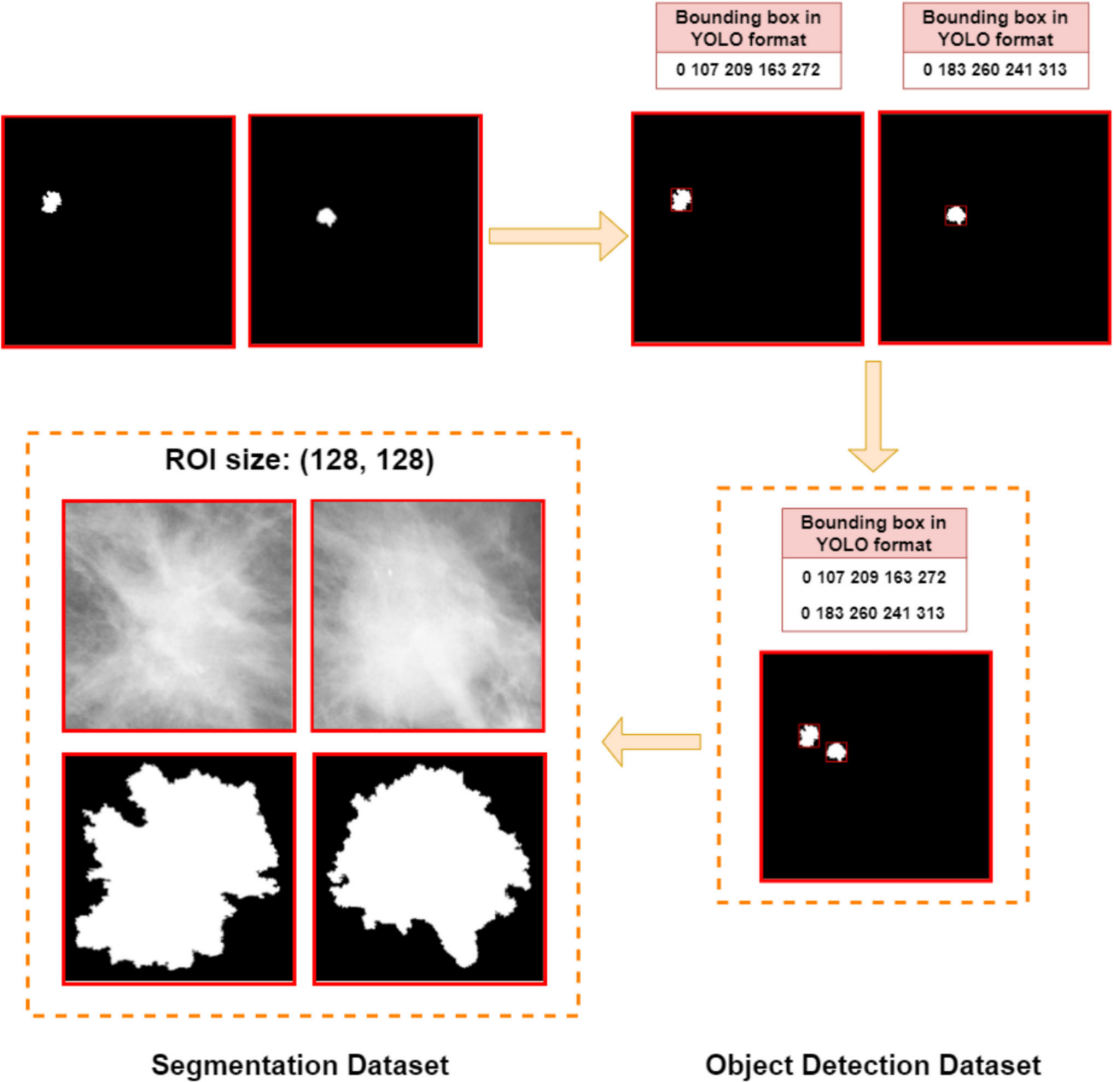
Final step of the data preprocessing. Masks from the same mammogram are combined into a single image, and bounding boxes are generated for each mass mask to serve as ground truth for object detection models. Bounding boxes are extracted before combining masks to ensure accuracy. The object detection dataset is created with combined masks and bounding boxes, while cropped images of each mass, perfectly aligned with their masks, are extracted for the segmentation dataset. Cropped images are padded to squares and resized to 128x128

In the original dataset, mammograms with multiple abnormalities have their corresponding masks stored as separate images. Furthermore, the dataset did not include any bounding boxes, providing only mass masks for each mammogram. The second part of the preprocessing evolves around combining masks from the same mammogram into a single image and generating bounding boxes surrounding each mass mask that will serve as ground-truth for the object detection models. Finally, cropped images are created for each mass to build the segmentation dataset.

As depicted in Fig. 3, the bounding boxes are extracted before combining the masks into a single image. Converting masks to bounding boxes is an easier and more error-resistant process for automated tools such as the PyTorch function *masks_to_boxes* [46] when a single mask per image is present. Moving on, after combining all masks and bounding boxes associated with the same mammogram into a single image and text file, respectively, the object detection dataset is created. Regarding the segmentation dataset, despite the CBIS-DDSM dataset offering pre-cropped images featuring mass ROIs, we chose to extract new cropped images for each mass utilizing the created bounding boxes, ensuring that the ROIs are perfectly aligned with their corresponding mask. As a final step, each cropped image is converted into a square shape through padding and resized to 128x128.

### Data Augmentation

Data augmentation has become a standard practice in many prior studies [6, 28]. This technique involves applying random yet realistic transformations to images, such as a horizontal flip, before feeding them into the neural network. Augmentation is performed only on the training and validation datasets, ensuring the integrity of the test set. Despite maintaining a consistent total image count, each training epoch introduces different variations of the original dataset, improving the performance of the model and reducing overfitting [47]. As regards the augmentation techniques that were used, we applied image rotation, cropping, scaling, and horizontal and vertical flips.

## Methodology

Our presented approach consists of two distinct methodologies. Firstly, we apply transfer learning (TL) on pre-trained Yolo models on the task of mass detection. The most lightweight YoloV5 [37] and YoloV8 [48] variations are examined since our aim is to minimize computational costs and especially inference time. Regarding the task of mass segmentation, by applying TL on pre-trained U-Net models, we try to evaluate the optimal number of parameters and architecture design for the task of medical segmentation based on their performance. Finally, we move on to training from scratch shallow neural networks with the aim of minimizing the trade-off between model performance and parameter efficiency.

### Detecting Masses in Mammograms Using the Yolo Architecture

For the task of detecting masses on mammograms, we decided to employ the YoloV5 architecture [37]. During the release of its 6th version, the YoloV5nano variation was published, which, in combination with the YoloV5small variation, provides two high-performing lightweight models with less than 10 M parameters and an CPU inference speed that is lower than 100 ms. Additionally, the YoloV5 architecture has been shown to have greater performance on the same task and dataset [35], in comparison with other detection techniques such as Faster R-CNN and Single-Shot detection, as well as the previous Yolo iteration, that is, YoloV3.

The developers of the YoloV5 model, Ultralytics, recently released the YoloV8 architecture [48]. Three primary architectural components characterize both the YoloV5 and YoloV8 models: the backbone, neck, and prediction head. The Yolo architecture utilizes cross stage partial networks [49] as its backbone, in order to extract features, which are then forwarded to the model’s neck, responsible for generating feature pyramids, that allow the model to detect the same object at various sizes and scales. Subsequently, the produced feature pyramid is fed into the model’s head to generate the final output, that is, a set of bounding boxes, class probabilities, and objectness scores for each detected object in the image.

The YoloV8 implements slight changes in the main three components, such as changing the number of filters in some convolutional layers and adding more skip connections. Additionally, it deviates from the anchor-based approach of YoloV5 by adopting an anchor-free model design. An anchor-based model, like YoloV5, uses a predefined set of anchor boxes of various sizes and aspect ratios. The model predicts the location and size of the bounding boxes relative to these anchor boxes. YoloV8 eliminates the use of anchor boxes. Instead, it directly predicts the center point and size of bounding boxes, reducing model complexity. The most lightweight architectures were selected for the YoloV8 model as well, namely YoloV8n and YoloV8s.

The YoloV5 models were trained with initial learning rate of 0.01, final learning rate of 0.1, momentum of 0.937, and weight decay of 0.0005 and stochastic gradient descent [50]. In the case of the YoloV8 models, the AdamW optimizer [51] was used with a learning rate of 0.002 and a momentum of 0.9. The IoU threshold was set to 0.2 for training and to 0.6 for testing. Finally, an early stopping procedure was applied, which stopped the models’ training after there was no improvement after 100 epochs. The maximum number of training epochs was set to 300.

### Efficient Mass Segmentation in Mammograms Using Lightweight Neural Networks

One of the most popular techniques for medical image segmentation is U-Net [27]. This architecture uses a CNN structure that resembles an encoder-decoder architecture and was designed for the task of medical image segmentation [13]. The encoder performs feature extraction while reducing the spatial resolution through convolutional and pooling operations, while on the other hand, the decoder up-samples the feature maps using deconvolutional layers, with the aim of generating a segmented mask that aligns with the original input image. The encoder and the decoder are connected through a series of skip connections that preserve information lost during down-sampling.

Transfer learning can be quite effective on small datasets, overcoming the obstacle of data scarcity that is quite evident in the domain of medical imaging. Additionally, lightweight pre-trained models such as MobileNet [52] can lead to significant performance and small inference time while maintaining a small training duration due to the good weight initialization from pre-training. In this work, we applied five different publicly available pre-trained models (through the segmentation-models PyTorch library [53]), namely, ResNet [54], MobileNetV2 [55], MobileNetV3 [56], EfficientNet [57], and VGG [29]. We tried to include variations of these architectures with different complexities in terms of number of parameters, in order to give us insights on the optimal complexity and network size for the specific task.

Moving on, our main goal is to create and train from scratch efficient neural network architectures that minimize network complexity, leading to a small inference time while maintaining a decent level of performance. In the first step, based on the validation results, we selected the best performing model (MobileNetV3 Small 0.75) from the TL procedure. We re-train this architecture from scratch without loading its pre-trained weights, aiming to evaluate the performance of U-Net in this task. Moving forward, we utilized the SegNet architecture [14] that also uses the encoder-architecture in combination with max-pooling layers for downsampling in the encoder. The main advantage of the SegNet architecture is that instead of skip connections that transfer the entire future map from the encoders to the corresponding decoders, only the max-pooling indices are transferred and used in the decoder layers in order to upsample their input. This sacrifices some performance but greatly reduces the number of parameters and memory consumption during inference, as well as the inference time, aligning with our goal of computationally efficient mass segmentation.

Moreover, we further improve the segmentation efficiency with the conversion of the convolutions performed by the model to depthwise separable convolutions (DSCs) [58]. This method factorizes a standard convolution to a depthwise and a pointwise convolution. A standard convolution performs the channel-wise and spatial-wise computations in one step, while a DSC splits this computation into two steps. During the depthwise step, a separate filter is applied to each input channel independently, while in the pointwise convolution, a 1x1 convolution is applied to combine the output channels produced by the depthwise convolution. DSCs are commonly used in edge devices since they provide an increased computational efficiency due to the reduced number of parameters, leading to a faster inference speed.

As regards the structure of the models, we experimented with different depths (number of encoder/decoder convolutional blocks), creating models SegNet3 and SegNet4 that employ the standard convolutions in combination with the SegNet architecture, having a total of 3 and 4 blocks, respectively. Models SegDSC3 and SegDSC4 are identical to the SegNet3 and SegNet4 models, but instead of the standard convolution, they utilize DSCs. Another three architectures were created and evaluated, namely SegDSC4_85, SegDSC4_90 and SegDSC4_95. Each of these networks reduces the number of filters per layer in model SegDSC4, by 85%, 90%, and 95%, respectively. Furthermore, we evaluate the performance of a traditional method, namely OTSU segmentation (threshold-based) [20].

**Table 1:** Mass detection: benchmark and experimental results

Architecture	Par/ters (M)	Flops (G)	CPU Inf/ce (ms)	CBIS-DDSM		INbreast	
				mAP@50	mAP@50-95	mAP@50	mAP@50-95
YoloV5n	1.9	4.5	45	50.3	20.1	68.2	31.5
YoloV5s	7.2	16.5	98	52.3	22.6	65.8	28.1
YoloV8n	3.2	8.7	80.4	54.0	23.0	65.6	30.8
YoloV8s	8.7	28.6	128.4	53.2	21.7	66.0	29.5

All models were trained using the Adam optimizer [59], an initial learning rate of 0.0001 for the pre-trained models, while for models that were trained from scratch, the initial learning rate was set to 0.01. A *ReduceLROnPlateau* scheduler [46] was applied during the training procedure of all models that reduced the learning rate by 40% with a patience of 5 epochs. An early stopping procedure was utilized that concluded training if there was no improvement after 25 epochs, while the maximum number of training epochs was set at 160.

### Evaluation Metrics

IoU is one of the most popular evaluation metrics both for object detection and segmentation tasks. For a prediction bounding box *A* and a ground truth bounding box *B*,

$$\begin{aligned} IoU = \frac{|A \cap B|}{|A \cup B|} \end{aligned}$$

An issue that limits IoU from being used as a loss function is that in cases where there is no intersection, IoU has no value and therefore no gradient. In order to address this issue, the loss function used by the Yolo architecture is Generalized Intersection over Union (GIoU) [60]. Given *C*, which is the smallest convex hull that encloses both *A* and *B*,

$$\begin{aligned} GIoU = \frac{|A \cap B|}{|A \cup B|} - \frac{|C \setminus (A \cup B)|}{|C|} = IoU - \frac{|C \setminus (A \cup B)|}{|C|} \end{aligned}$$

We evaluate the performance of the object detection models on the test datasets using mean average precision (mAP) [62]:

$$\begin{aligned} mAP = \frac{1}{n} \sum _{k=1}^n AP_k \end{aligned}$$

where *n* is the number of classes (that is one for the task of mass detection) and $AP_k$ is the average precision for class *k*. When the prediction IoU threshold is set to 0.5, the metric *mAP*@0.5 is computed. We also consider *mAP*@05 : 0.95 that is computed for various IoU thresholds from 0.5 to 0.95 with a step of 0.05.

As regards the segmentation models, during our experiments, we utilized the Dice similarity loss function [61], which, given the ground truth segmentation mask *Y* and the predicted segmentation mask *X*, is defined as

$$\begin{aligned} DiceLoss = 1 - \frac{2|X \cap Y|}{|X| + |Y|} \end{aligned}$$

For evaluation purposes, we report the Dice coefficient and IoU (with a threshold of 0.5) on the test datasets.

## Experimental Results

In this section, we present the evaluation results of the detection and segmentation models on the test datasets. Since our aim is to develop a solution that minimizes the computational costs (e.g., memory consumption and speed) while maintaining a decent performance, we provide a detailed benchmark for all of the models that were used in this paper. All models were implemented on PyTorch and trained on a NVIDIA GeForce RTX 3080 Ti GPU with 12 GB memory.

### Mass Detection Results

**Fig. 4:**
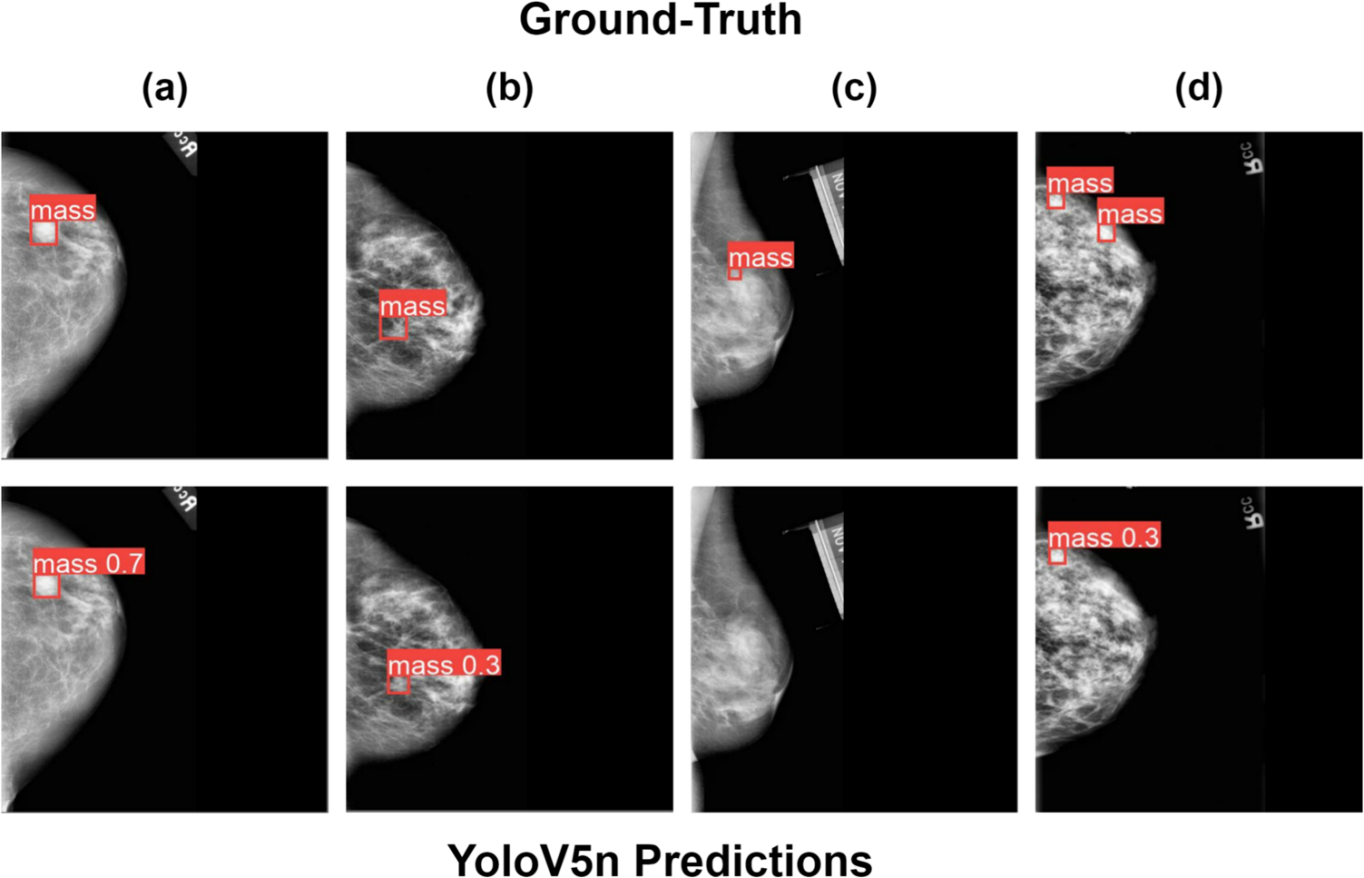
Execution samples of the YoloV5n model on the CBIS-DDSM test dataset: **a** a success case in which the model correctly detects the mass with a high confidence of 0.7, **b** the model correctly detects the mass but with a low confidence of 0.3, **c** a failure case in which the model makes no prediction and **d** the model correctly detects on one of the masses, but it does not detect the second one

The evaluation results of the mass detection models on the CBIS-DDSM and INbreast test datasets are summarized at Table 1. The benchmarking information was provided by the developers of the YoloV5 [37] and YoloV8 [48] models. The YoloV5 nano version is the most efficient model out of all the Yolo architectures, with less than 2 M parameters and an inference time of 45 ms. Additionally, even though it does not perform as well as the other models on the CBIS-DDSM dataset, it showcases significant generalization capabilities by achieving the best performance on the INbreast test dataset, with an mAP of about 70%. Hence, this model was selected for integration into the final two-stage model due to its decent performance while having the lowest inference speed. In the case of the YoloV8 models, they have more parameters and slower inference time than their YoloV5 counterparts. Even though both of them perform slightly better on the CBIS-DDSM dataset, on the INbreast data, most of the models have similar performance (with the exception of YoloV5n). Figure 4 provides some success and failure cases of the selected detection model YoloV5n.

### Mass Segmentation Results

**Table 2:** Mass segmentation: benchmark

Architecture	Par/ters (M)	Size (MB)	Flops (G)	Training		Inference	
				Time (m)	Memory (MB)	Time (ms)	Memory (MB)
ResNet18	14	57	1.35	2.1	528.5	59.98	80.11
MNetV2	6.6	26	0.84	3.0	707.5	66.28	158.7
EfficientNet-b0	6.2	9.1	0.633	4.7	785.8	80.51	157.9
EfficientNet-b1	8.7	9.2	0.636	7.0	1003	107.0	168.3
EfficientNet-b2	10	9.6	0.64	8.3	1056	112.9	173.6
EfficientNet-b3	13	10	0.67	15.4	1315	126.8	187.2
VGG13	18	73	4.8	3.7	838.9	43.82	130.9
MNetV3-S_1	3.0	12	0.639	5.9	344.4	43.09	143.5
MNetV3-S_.75	2.8	11	0.61	5.3	346.9	52.17	142.7
MNetV3-L_1	5.1	20	0.76	3.7	506.1	55.26	152.0
MNetV3-L_.75	4.8	19	0.70	6.0	517.4	62.41	151.0
MNetV3-S_.75 (s)	2.8	11	0.61	2.0	346.9	51.49	142.7
SegNet3	2.2	9.1	4.9	5.5	1710	31.89	82.95
SegNet4	14	56	7.93	6.4	1950	43.43	131.2
SegDSC3	0.271	1	0.62	3.8	1490	30.06	177.4
SegDSC4	1.6	6	0.96	4.9	1653	37.56	183.4
SegDSC4_85	0.422	0.17	0.03	12.6	355.7	21.11	139.5
SegDSC4_90	0.020	0.08	0.01	8.5	292.8	20.79	140.5
SegDSC4_95	0.006	0.02	0.007	10.3	221.4	19.69	137.6

**Table 3:** Mass segmentation: experimental results

Architecture	CBIS-DDSM		INbreast	
	IoU	Dice	IoU	Dice
ResNet18	82.4	90.1	78.4	87.5
MNetV2	82.3	90.1	78.5	87.6
EfficientNet-b0	82.9	90.5	78.5	87.6
EfficientNet-b1	83.0	90.4	78.8	87.8
EfficientNet-b2	82.5	90.3	78.4	87.6
EfficientNet-b3	82.7	90.4	78.1	87.4
VGG13	82.4	90.2	78.2	87.4
MNetV3-S_1	82.5	90.2	78.9	87.8
MNetV3-S_.75	81.6	89.7	77.0	86.7
MNetV3-L_1	81.7	89.8	77.6	87.1
MNetV3-L_.75	81.6	89.7	77.0	86.7
MNetV3-S_.75 (s)	82.8	90.4	79.0	88.0
SegNet3	79.2	87.5	76.0	85.5
SegNet4	82.0	89.3	78.5	87.1
SegDSC3	78.9	87.5	75.9	85.5
SegDSC4	79.5	87.9	75.8	85.5
SegDSC4_85	80.7	69.3	77.6	69.4
SegDSC4_90	81.0	89.4	77.3	87.0
SegDSC4_95	79.3	88.4	75.6	85.8
OTSU	52.2	32.0	50.0	33.6

Table 2 includes the benchmarking of all the segmentation models. The number of parameters and the size of the model were computed using the Python library torchinfo [62]. The number of floating-point operations per second (Flops) was estimated for the forward pass of each model (in GigaFlops), using Python’s library thop [63]. The training of each model is measured in minutes. Note that because of the early stopping procedure, the models are trained for a different number of epochs. The memory consumption during training and testing, as well as the inference speed, is computed using PyTorch’s profiler function. Inference speed and memory consumption are calculated using a batch size of 1, while for training, the whole test dataset is used. Finally, Table 3 contains the evaluation results for all the pre-trained models, the models that were trained from scratch and the OTSU method.

As regards the evaluation of the pre-trained models (first 11 entries of Tables 2 and 3), they achieve a high level of performance, with 8 out of 11 having a Dice score of about 90% and 87.5% at the CBIS-DDSM and the INbreast dataset, respectively. When both performance and efficiency are considered, the small MobileNetV3 architecture (MNetV3-S_1) stands out as it has the fastest inference speed out of all pre-trained models at 43 ms. Moreover, it maintains the highest performance among pre-trained models on the INbreast dataset, with an IoU of 78.9% and a Dice score of 87.8%. Moving on, the MobileNetV3 architecture MNetV3-S_.75, which had the best performance on the validation data out of the pre-trained models, was trained from scratch without loading the pre-trained weights. Interestingly, the performance of the model is slightly better in both datasets in comparison with its pre-trained counterpart, showing evidence that the method of TL is not required for the specific task.

The final 7 entries of Tables 2 and 3 concern the proposed architectures that were trained from scratch. Considering that all of the pre-trained U-Net models have 5 encoder/decoder blocks, it is clear that the SegNet architecture leads to a high number of parameters, since even with 4 blocks (SegNet4), it leads to a total of 14 M parameters (5 times more than MNetV3-S_.75). Having said that, SegNet models have impressive performance during inference, similar to that of lightweight models. For instance, SegNet4 achieves an IoU of 82% on the CBIS-DDSM test dataset (0.8% lower than MNetV3-S_.7), with an inference speed of 43 ms (similar to MNetV3-S_.7) and a memory consumption of 131 MB (lower than MNetV3-S_.7).

The intergration of DSCs into the SegNet models greatly reduces the number of parameters (about 12% for both SegDSC3 and SegDSC4) and, consecutively, the inference time (by 4 and 6 ms) but leads to a drop in performance for SegDSC4, while SegDSC3 retains the performance levels of SegNet3. In the final step, models SegDSC4_85, SegDSC4_90, and SegDSC4_95 reduce the number of filters in the DSC layers of SegDSC4. This leads to the development of neural networks with a very small number of parameters; SegDSC4_90 consists of 20.000 parameters, while SegDSC4_95 has 6.000 parameters. On the one hand, SegDSC4_85 fails to produce acceptable results, having a Dice score of about 70% on both test datasets. On the other hand, both SegDSC4_90 and SegDSC4_95 achieve decent performance on both metrics and testing datasets. SegDSC4_90 leads to an IoU of 81% and a Dice score of 89.4% (only 1.8% and 1% lower than MNetV3-S_.7), while maintaining an inference speed of about 21 ms, making it twice as fast as MNetV3-S_.7. Finally, due to the fact that neural networks of such low complexity managed to produce high levels of performance, we opted to evaluate simple segmentation techniques such as OTSU thresholding. Based on the results of Table 3, it is clear that this method can not achieve a satisfying level of performance, with a Dice score of about 33% and an IoU of approximately 51% on both testing datasets.

**Fig. 5:**
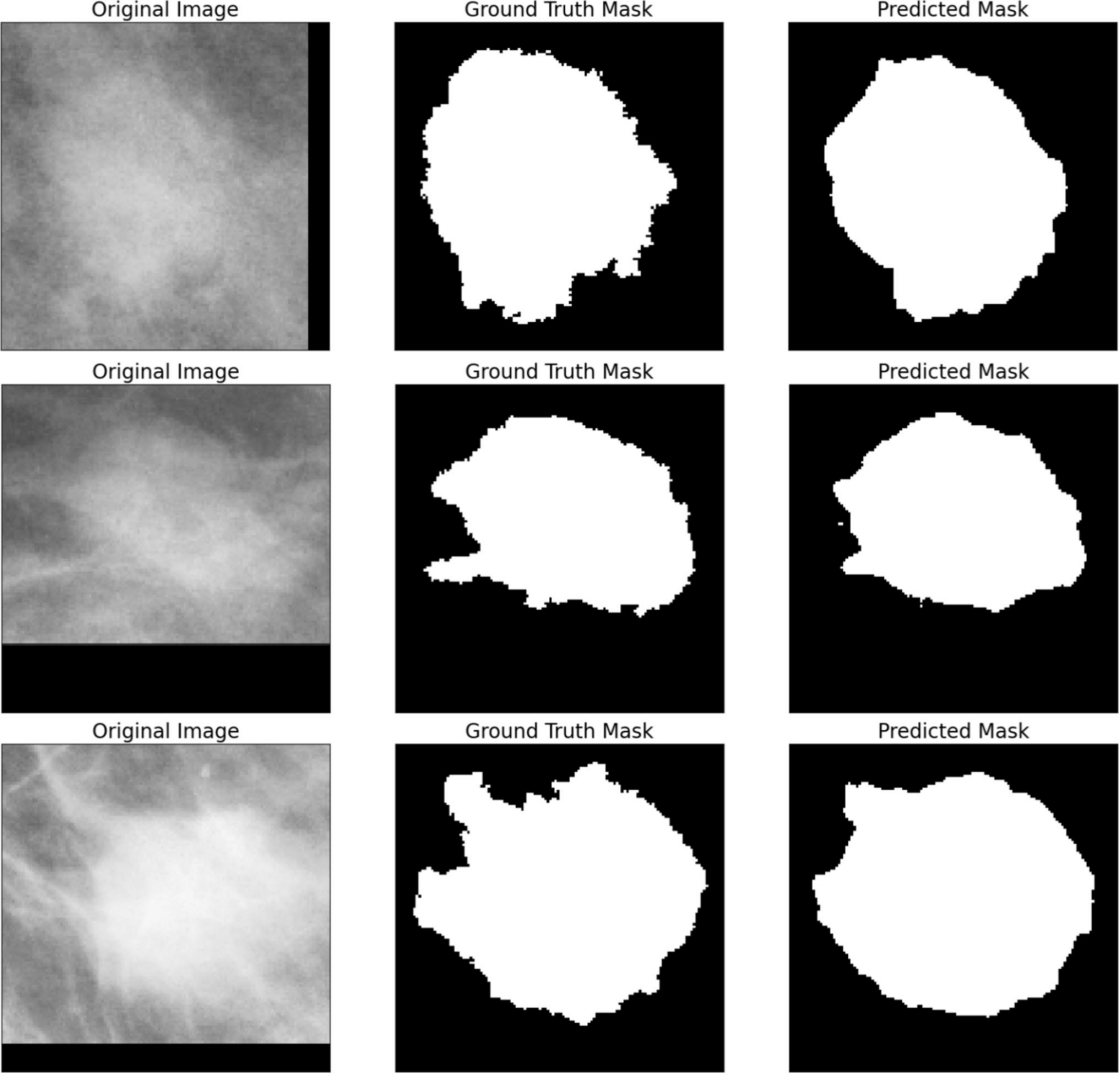
Three samples of the segmentation masks produced by model SegDSC4_90 on mammograms of the CBIS-DDSM test data

It is important to note that the reported inference speed currently utilizes GPU acceleration. Given the objective for the segmentation model to run in browsers without GPU support, the model will undergo conversion from PyTorch format, potentially leading to a significant increase in final inference speed. Hence, the minimization of the inference time during the development phase is crucial. Considering the balance between performance and speed achieved by model SegDSC4_90, making it well-suited for the intended browser-based deployment scenario, it is selected as the segmentation component of our two-stage model. Figure 5 illustrates the predictions produced by the SegDSC4_90 model on three mammograms of the CBIS-DDSM testing data.

### Comparison of the Proposed Architecture and State-of-the-Art Methods

In this section, we make comparisons of our proposed architectures with state-of-the-art methods. For the mass detection task, since our results demonstrated that YoloV5 outperforms YoloV8, we focused on comparisons with earlier Yolo iterations and other detectors, such as Faster R-CNN and Single-Shot Detector. Table 4 presents the parameter count alongside the performance of various methods, evaluated in terms of mAP@50 on the CBIS-DDSM and INbreast datasets. It is important to note that the methods proposed by Su et al. [35] and in our study utilized INbreast exclusively as a testing set, whereas the methods developed by Aly et al. [64] were both trained and tested on INbreast, which, as expected, resulted in higher performance.

**Table 4:** Performance comparison of the proposed YoloV5n with state-of-the-art methods

Reference	Method	Par/er count (M)	Dataset	mAP@50
Our work	YoloV5n	1.9	CBIS-DDSM	50.3
			INbreast	68.2
Aly et al. [64]	YoloV1	62.0	INbreast	48.8
	YoloV2	50.0	INbreast	69.5
Su et al. [35]	YoloV3-tiny	8.9	CBIS-DDSM	53.0
			INbreast	45.1
	YoloV3	61.9	CBIS-DDSM	60.0
			INbreast	56.5
	YoloV3-spp	63.0	CBIS-DDSM	63.0
			INbreast	58.3
	Faster R-CNN	$\ge$40.0	CBIS-DDSM	50.6
			INbreast	47.0
	Single-Shot Detection	34.0	CBIS-DDSM	48.3
			INbreast	42.1

**Table 5:** Performance comparison of the proposed SegDSC4_90 with state-of-the-art methods

Reference	Method	Par/er count (M)	Dataset	Dice score (%)	IoU score (%)
Our work	SegDSC4_90	0.02	CBIS-DDSM	89.4	81.0
			INbreast	87.0	77.3
Cho et al. [6]	U-net	1.8	INbreast	80.0	−
Tsochatzidis et al. [65]	U-net+	22.5	CBIS-DDSM	72.2	56.5
Alexey et al. [28]	VGG U-net	23.7	CBIS-DDSM	81.3	65.2
Baccouche et al. [7]	Connected-UNets	20	CBIS-DDSM	87.0	77.0
			INbreast	95.1	90.7
	Connected-AUNets	12.5	CBIS-DDSM	87.9	78.8
			INbreast	94.8	90.2
	Connected-ResUNets	20.7	CBIS-DDSM	89.5	80.0
			INbreast	95.2	91.0

It is evident that most state-of-the-art methods are not lightweight, with parameter counts exceeding 30 million. The most lightweight among the methods, Yolov3-tiny, consists of 9 million parameters and achieves 3% better performance than our proposed YoloV5n on the CBIS-DDSM dataset. However, on the INbreast dataset, our proposed method outperforms YoloV3-tiny by more than 20%, despite having over three times fewer parameters. The best overall performance is achieved by YoloV3-spp, with 63% on CBIS-DDSM and 58.3% on INbreast, utilizing a total of 63 million parameters. It is worth noting that our method has the highest performance (68.2%) on the INbreast dataset, considering that the YoloV2 method (with an mAP of 69.52%) was trained on a split of that dataset.

Regarding the segmentation models, Table 5 provides a performance comparison between our proposed method and other U-Net-based deep learning approaches. Notably, all the methods included in the comparison were trained on both the CBIS-DDSM and INbreast datasets, whereas in our case, the INbreast dataset was exclusively used for testing.

**Fig. 6:**
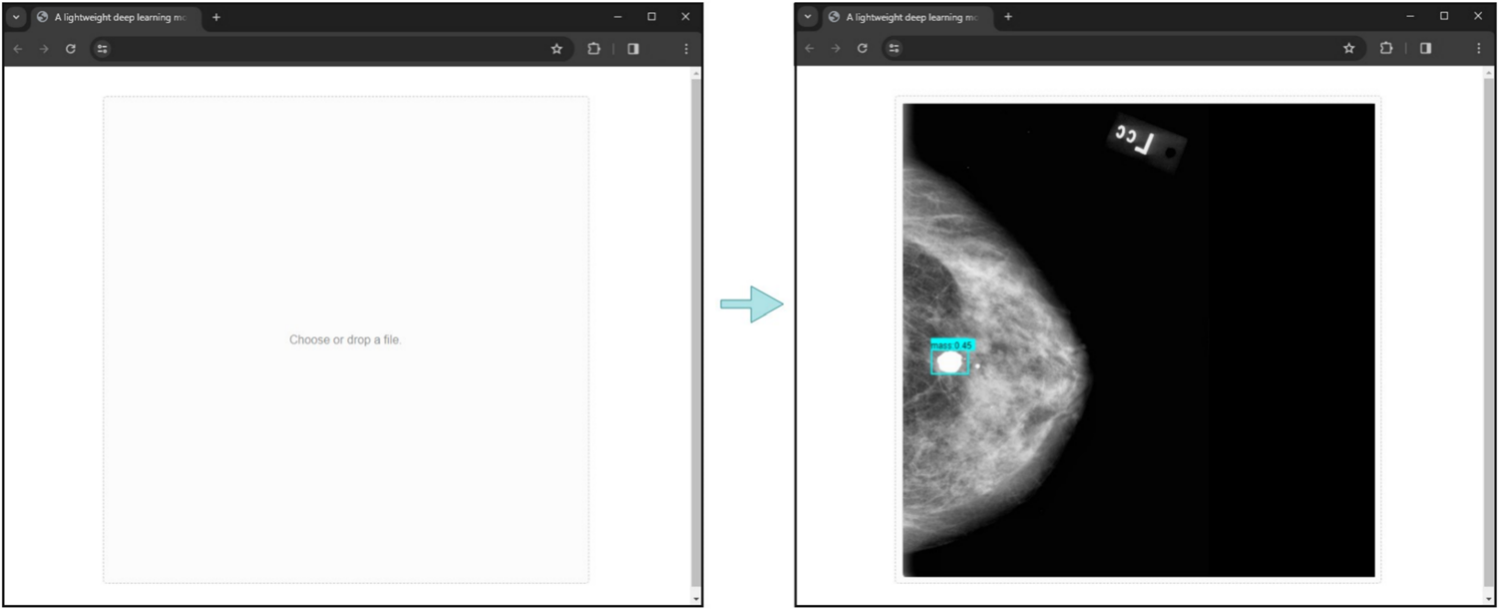
User interface of the web application integrating the two-stage model using TensorFlow.js: the initial state of the application that prompts the user to upload a mammogram (left), the bounding box and the segmentation mask of the detected mass are showcased on top of the original mammogram (right)

Our method achieves a Dice score of 89.4% and an IoU score of 81% on the CBIS-DDSM dataset, outperforming nearly all state-of-the-art methods. The only exception is the Connected-ResUNets, which demonstrate comparable performance with a Dice score of 89.52% and an IoU score of 80.02%. For the INbreast dataset, all Connected-UNets proposed by Baccouche et al. [7] surpass our method in performance. However, it is important to note that their models were trained using the INbreast dataset, which likely explains this disparity. Overall, our proposed SegDSC4_90 achieves performance that is comparable to or better than state-of-the-art neural networks with over 10 million parameters, showcasing its efficiency and effectiveness.

### Deployment of the Two-Stage Model into a Browser Application

Figure 6 illustrates the current version of our web application that integrates the proposed two-stage model. The application runs directly within the user’s browser, reducing latency since there is no need for server-side response exchanges. Additionally, since the data are not uploaded to a server, privacy is enhanced, which is crucial when it comes to medical data. The first step for the development of the application is to convert both models to the appropriate format. Since the framework TensorFlow.js [15] is used in order to run the PyTorch models on the browser, they are converted to the Open Neural Network Exchange format [66] and then to the TensorFlow format. Moving on, the interface of the application is developed using React. Current functionalities allow the user to provide a mammogram, which will be passed to the object detection model. Using the produced bounding boxes, cropped images centered around the detected masses serve as input to the segmentation model in order to extract the segmentation mask, as depicted in Fig. 6.

## Conclusions and Future Challenges

Our work resulted in the development of a two-stage model designed to address the task of breast cancer detection through mass identification and segmentation. We developed a robust data preprocessing pipeline that cleans and enhances the quality of the medical dataset through cropping, flipping, padding, resizing, and advanced enhancement techniques. The proposed solution utilizes the YoloV5n model in order to firstly detect masses and then makes use of a lightweight SegNet model, combined with DSCs, in order to extract the segmentation mask. The two-stage model can handle cases of multiple masses per mammogram.

The main advantage of the presented approach is the fact that it achieves good levels of performance while being extremely efficient in terms of size, inference speed, and memory consumption. The object detection model is the most lightweight YoloV5 architecture, producing an mAP@50 equal to 50.3% on the CBIS-DDSM dataset and 68.2% on the INbreast. In the case of the segmentation model, with a structure of only 20.000 parameters, attains high-performance levels on the CBIS-DDSM (81.0% IoU, 89.4% Dice) and INbreast (77.3% IoU, 87.0% Dice) datasets, having an inference time of just 21 ms. This facilitates real-time detection and segmentation results on mammograms with minimal latency, allowing integration of our proposed solution into a browser-based web application. Crucially, this allows our application to uphold strict privacy standards around medical information since the data never leave the user’s computer.

Future challenges include the extension of our work in order to include more functionalities for the users of our application, such as tumor classification or automated reporting based on the mammogram and the detected masses. Combining the loss functions of each task and making the system end-to-end is a significant future challenge. Finally, more advanced data augmentation techniques such as diffusion models [67] are worth exploring since they can further improve the performance of both models.

## Data Availability

No new data were created or analyzed in this study. Data sharing is not applicable to this article.
